# Vorinostat enhances the anticancer effect of oxaliplatin on hepatocellular carcinoma cells

**DOI:** 10.1002/cam4.1278

**Published:** 2017-12-13

**Authors:** Bo Liao, Yingying Zhang, Quan Sun, Ping Jiang

**Affiliations:** ^1^ Department of Hepatopancreatobiliary Surgery Zhongnan Hospital of Wuhan University Wuhan China; ^2^ Intensive Care Unit Zhongnan Hospital of Wuhan University Wuhan China

**Keywords:** BRCA1, combination, hepatocellular carcinoma, oxaliplatin, vorinostat

## Abstract

Oxaliplatin‐based systemic chemotherapy has been proposed to have efficacy in hepatocellular carcinoma (HCC). We investigated the combination of vorinostat and oxaliplatin for possible synergism in HCC cells. SMMC7721, BEL7402, and HepG2 cells were treated with vorinostat and oxaliplatin. Cytotoxicity assay, tumorigenicity assay in vitro, cell cycle analysis, apoptosis analysis, western blot analysis, animal model study, immunohistochemistry, and quantitative PCR were performed. We found that vorinostat and oxaliplatin inhibited the proliferation of SMMC7721, BEL7402, and HepG2 cells. The combination index (CI) values were all <1, and the dose‐reduction index values were all greater than 1 in the three cell lines, indicating a synergistic effect of combination of the two agents. Coadministration of vorinostat and oxaliplatin induced G2/M phase arrest, triggered caspase‐dependent apoptosis, and decreased tumorigenicity both in vitro and in vivo. Vorinostat suppressed the expression of BRCA1 induced by oxaliplatin. In conclusion, cotreatment with vorinostat and oxaliplatin exhibited synergism in HCC cells. The combination inhibited cell proliferation and tumorigenicity both in vitro and in vivo through induction of cell cycle arrest and apoptosis. Our results predict that a combination of vorinostat and oxaliplatin may be useful in the treatment of advanced HCC.

## Introduction

Hepatocellular carcinoma (HCC) remains a major health conundrum worldwide 1, 2. Although it is considered resistant to conventional chemotherapeutic agents 3, 4, 5, many reports have demonstrated that systemic chemotherapy containing oxaliplatin exerted significant anticancer effect on HCC 6, 7, 8, 9, 10, 11.

Oxaliplatin was first discovered in Japan in 1976 12, 13 and approved for clinical treatment in 1994 14. As a second‐generation platinum drug, it was initially used in metastatic colorectal cancer and afterward found to be effective in combination with other drugs for alimentary canal cancers 15. Its full chemical name is oxalato(trans‐L‐1,2‐diaminocyclohexane)platinum because of the presence of the 1,2‐diaminocyclohexane (DACH) carrier ligand and oxalate “leaving group” 16. The DACH carrier ligand renders it more effective than its analogs cisplatin and carboplatin. Its main mechanism of action is via the formation of DNA adducts 17. Once inside the body, DACH ligand is transformed into a DACH platinum compound that binds to the nitrogen atom (N7) of guanine, followed by formation of transient monoadducts and stable diadducts 15. In addition to DNA intrastrand crosslinks, oxaliplatin can induce DNA interstrand crosslinks and DNA‐protein crosslinks that cause DNA lesions, inhibition of DNA synthesis and repair, cell cycle arrest, and ultimately apoptosis 15, 18.

Epigenetic alterations occur in a variety of cancers including HCC. Among them, histone acetylation plays a crucial role in tumor initiation and development 19, 20, 21, and the use of histone deacetylase (HDAC) inhibitors has been considered as a novel approach for the treatment of numerous malignancies 22, 23, 24, 25, 26. A number of HDAC inhibitors are currently being used in experimental studies and clinical trials targeted to hematological malignancies and solid tumors. For solid tumors, HDAC inhibitors have to be combined with other chemotherapy agents to fully exert their antitumor activity 27.

The combination of HDAC inhibitors and oxaliplatin has been reported in gastric, pancreatic, and colorectal cancers 28, 29, 30, 31, 32, 33, but the effect on HCC remains unknown. The HDAC inhibitor vorinostat was first approved by the FDA for the therapy of refractory cutaneous T‐cell lymphoma 34, 35 and has been widely used in the treatment of many solid tumors.

In our study, we investigated the combination of vorinostat and oxaliplatin in HCC cell lines in vitro and in vivo and found that the combination of these two drugs had a synergistic effect on HCC.

## Materials and Methods

### Cell culture and agents

Human hepatocellular carcinoma cell lines SMMC7721 and BEL7402 and the human hepatoma cell line HepG2 were purchased from China Center for Type Culture Collection (CCTCC, Wuhan, China). All cell lines were cultured in high‐glucose Dulbecco's modified Eagle's medium (DMEM, Gibco, USA) containing 10% fetal bovine serum (FBS, Gibco, USA). Vorinostat, oxaliplatin, and DMSO were purchased from Sigma‐Aldrich (St. Louis, MO, USA). Vorinostat and oxaliplatin were dissolved in DMSO.

### Cell viability assay, CI analysis, and dose‐reduction index analysis

BEL7402 (2 × 10^3^ cells per well), SMMC7721 (1.5 × 10^3^ cells per well), and HepG2 (1 × 10^3^ cells per well) were plated in 96‐well plates and treated with increasing concentrations of vorinostat or oxaliplatin for 48 h to generate a cell viability curve. Cell Counting Kit‐8 (CCK‐8, Dojindo, Japan) reagent was added to plates for 2 h and the optical density (OD) values were measured at 450 nm. The half maximal inhibitory concentration (IC_50_) was calculated by SPSS software. Next, the cells were treated with increasing concentrations of vorinostat and oxaliplatin in a fixed ratio of IC50 of the two agents for 48 h to form a new cell viability curve. Chou previously explained the CI model 36, 37. Based on his theory, the CI value in our study was calculated as: CI = *a*/*A* + *a*′/*A*′, where *A* and *A*′ are the concentrations of single drug required to inhibit *x*% of cells, and a and a′ are the respective drug concentrations of the combination that inhibits *x*% of cells. A CI < 1 indicates synergism of the combination. In his papers, Chou mentioned the dose‐reduction index (DRI) determined by the fold reduction in the dose of the agents compared with the dose of agent used in isolation at a fixed effect level 36, 37. DRI > 1 indicates a reduced dose and reduced toxicity after combination treatment. DRI for each corresponding drug was calculated as: DRI (*A*) = *A*/*a* or DRI (*A*′) = *A*′/*a*′.

### Western blotting

Immunoblotting was performed as described previously 38. The primary antibodies against acetylated histone H3 and *β*‐actin were purchased from Santa Cruz Biotechnology (Santa Cruz, CA, USA), and antibodies against cleaved‐caspase 9, cleaved‐caspase 7, PARP, and BRCA1 were purchased from Cell Signaling Technology (Beverly, MA, USA). All horseradish peroxidase (HRP) secondary antibodies were purchased from Jackson Immuno Research Laboratories (West Grove, PA, USA).

### Colony formation assay and soft agar assay

Colony formation assay was carried out as described previously 39. HepG2 (500 cells/well) and SMMC7721 (300 cells/well) were seeded in six‐well plates and treated with vorinostat and/or oxaliplatin for 48 h. Media were refreshed every other day. The wells were stained with crystal violet (Sigma‐Aldrich, USA) and their images were acquired at day 14. The numbers of colonies were counted and analyzed by Alpha Innotech Imaging system (Alphatron Asia Pte Ltd, Singapore). Soft agar assay was performed as previously reported 40. HepG2 (5000 cells/well) and SMMC7721 (5000 cells/well) were plated in six‐well plates and treated with culture media containing vorinostat and/or oxaliplatin, which was replaced every 2 days. At day 14, the colonies were counted and analyzed as described above.

### Cell cycle and apoptosis analysis

The flow cytometry analysis was carried out as described previously 41. For cell cycle analysis, HepG2 and BEL7402 cells were treated with vorinostat and/or oxaliplatin for 48 h. A total of 1 × 10^6^ cells per sample were analyzed using FACSAria Cell Cytometer (BD Biosciences, San Jose, CA, USA). For apoptosis analysis, 1 × 10^5^ cells per well were tested. All data were analyzed using CellQuest software (BD Biosciences).

### Xenograft tumorigenicity assay

The animal studies were performed as previously described 39, 40. All procedures performed in animal studies were approved by the Committee on the Ethics of Animal Experiments of Zhongnan Hospital, Wuhan University. HepG2 cells were subcutaneously injected into the mice. Drug treatment started when the tumors reached 100 mm^3^ in size. Vorinostat (25 mg·kg^−1^) was injected intraperitoneally everyday, and oxaliplatin (5 mg·kg^−1^) was injected intraperitoneally twice a week. Subcutaneous tumor xenografts were removed and conserved for subsequent analysis.

### Immunohistochemistry analysis

Immunohistochemistry was performed as previously described 39, 40. Ki‐67 primary antibody was obtained from Dako (Golstrup, Denmark). The paraffin‐embedded sections of the xenografts were detected using the TUNEL assay kit (R&D Systems, Minneapolis, MN, USA) for apoptosis analysis.

### Real‐time quantitative PCR analysis

PCR was performed as described previously 42. The primer sequences for BRCA1 were as follows: sense 5′‐GGCTATCCTCTCAGAGTGACATTT‐3′, anti‐sense 5′‐GCTTTATCAGGTTATGTTGCATGG‐3′. Expression of *β*‐actin mRNA was used as an internal control for normalization. Results were calculated as fold induction relative to *β*‐actin.

### Transient RNA interference

Small interfering RNA (siRNA) duplexes targeting human BRCA1 sequences and a scrambled siRNA were designed as described previously 43, 44. All siRNAs were synthesized by Ribobio (Guangzhou, China). Transfection of the siRNA duplexes was performed using jetPRIME (Polyplus‐transfection SA, Illkirch, France) according to the manufacturer's instructions.

### Statistical analyses

Data analyses were carried out using GraphPad Prism 5.0 (La Jolla, CA, USA) or SPSS 13.0 (Chicago, IL, USA). All of the experiments were performed at least three independent times. The results were presented as mean ± SEM. Comparisons between the different groups were analyzed by one‐way ANOVA with *P* < 0.05 considered statistically significant.

## Results

### Vorinostat and oxaliplatin attenuate the growth of HCC cells

**Figure 1:**
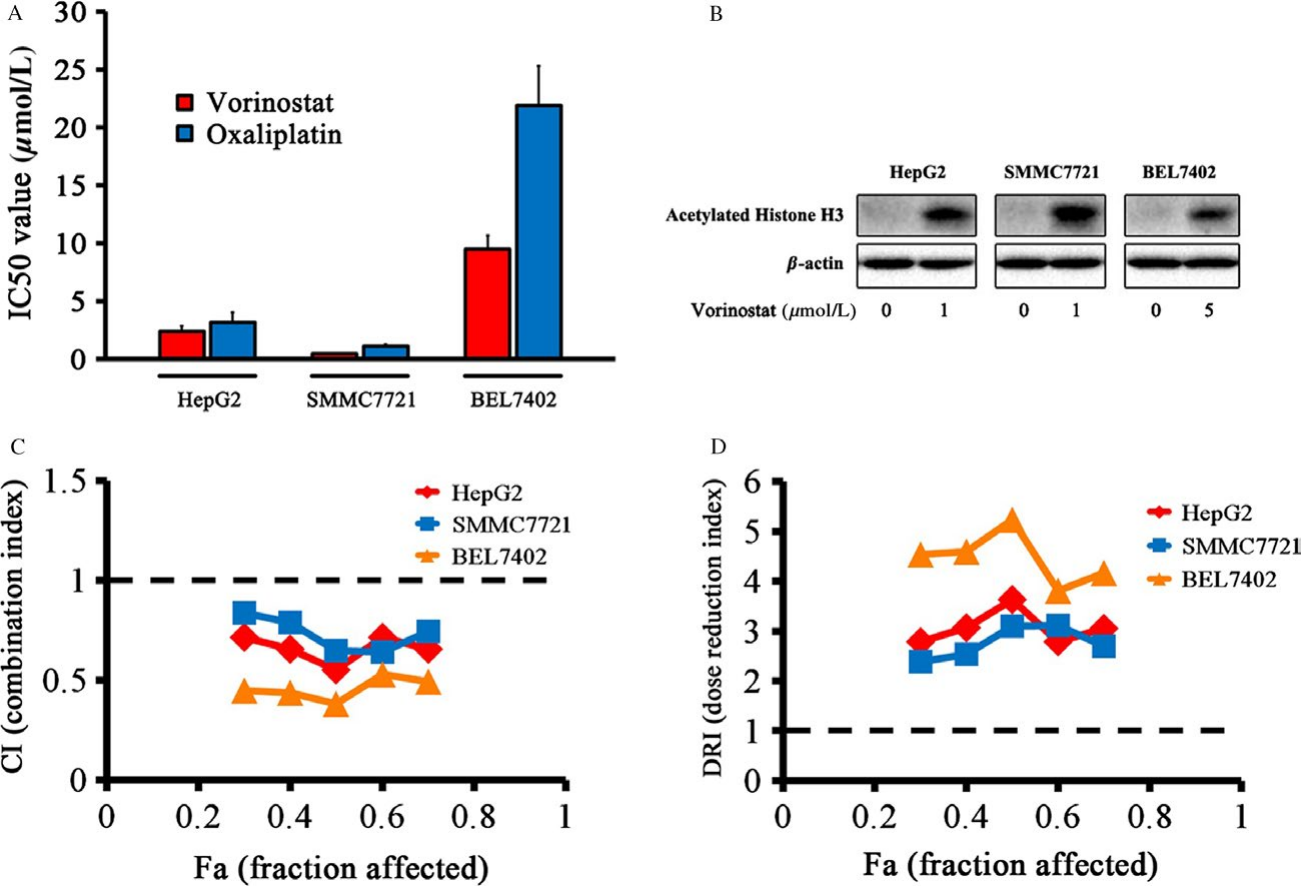
Vorinostat and oxaliplatin attenuated the proliferation of HCC cell lines. (A) Cytotoxicity assay. HepG2, SMMC7721, and BEL7402 cells in 96‐well plates were treated with different concentrations of vorinostat and oxaliplatin for 48 h and cell viability was detected by the Cell Counting Kit‐8 (CCK‐8) assay. The half maximal inhibitory concentration (IC50) was calculated by SPSS software. (B) HepG2 and SMMC7721 cells were treated with 1 *μ*mol/L vorinostat and BEL7402 cells were treated with 5 *μ*mol/L vorinostat. The expression of acetylated Histone H3 was detected by western blotting. (C) Combination index (CI) values of HepG2, SMMC7721, and BEL7402 cells at different fractions affected. (D) Dose‐reduction index (DRI) values of HepG2, SMMC7721, and BEL7402 cells at different fractions affected. All experiments were performed at least three independent times.

We first investigated the effect of vorinostat or oxaliplatin alone on cell growth in three HCC cell lines. HepG2, SMMC7721, and BEL7402 cells were cultured with different concentrations of vorinostat or oxaliplatin for 48 h. Both vorinostat and oxaliplatin inhibited proliferation of the three cell lines. The IC50 values for vorinostat and oxaliplatin are shown in Figure 1A and Table 1.

**Table 1:** The IC50 values for vorinostat and oxaliplatin in HCC cell lines

Cell line	IC50 values of drugs (*μ*mol/L)	
	Vorinostat	Oxaliplatin
HepG2	2.39 ± 0.48	3.17 ± 0.87
SMMC7721	0.47 ± 0.08	1.14 ± 0.14
BEL7402	9.49 ± 1.18	21.92 ± 3.38

### Vorinostat increases the acetylation of histone H3 in HCC cells

The effect of vorinostat on the acetylation of histone H3 was tested in HCC cells. The results demonstrated that vorinostat treatment for 48 h increased the acetylation level of histone H3 in HepG2, SMMC7721, and BEL7402 cell lines (Fig. 1B).

### Vorinostat increases the antiproliferative efficacy of oxaliplatin in HCC cells

Combination treatment with vorinostat and oxaliplatin attenuated cell growth more than either drug used in isolation in the three cell lines. The CI values in HepG2, SMMC7721, and BEL7402 cells were all less than 1, which indicated a synergistic effect after combination treatment with the two drugs (Fig. 1C). The DRI values of the two drugs were greater than 1 in all three cell lines (Fig. 1D). The CI and DRI values for coadministration of vorinostat and oxaliplatin are listed in Table 2.

**Table 2:** CI and DRI values after combination treatment with vorinostat and oxaliplatin

		Cell line		
		HepG2	SMMC7721	BEL7402
CI values at % fraction affected	30%	0.72 ± 0.03	0.84 ± 0.04	0.45 ± 0.08
	40%	0.66 ± 0.06	0.79 ± 0.02	0.44 ± 0.03
	50%	0.55 ± 0.06	0.65 ± 0.05	0.38 ± 0.03
	60%	0.72 ± 0.03	0.64 ± 0.04	0.53 ± 0.07
	70%	0.67 ± 0.05	0.75 ± 0.05	0.49 ± 0.10
DRI values at % fraction affected	30%	2.79 ± 0.13	2.40 ± 0.14	4.54 ± 0.79
	40%	3.06 ± 0.29	2.53 ± 0.06	4.59 ± 0.32
	50%	3.64 ± 0.38	3.10 ± 0.31	5.24 ± 0.43
	60%	2.79 ± 0.10	3.12 ± 0.23	3.81 ± 0.49
	70%	2.98 ± 0.21	2.69 ± 0.25	4.17 ± 0.81

CI, combination index; DRI, dose‐reduction index.

### Vorinostat and oxaliplatin decrease the tumorigenicity of HCC cells in vitro

**Figure 2:**
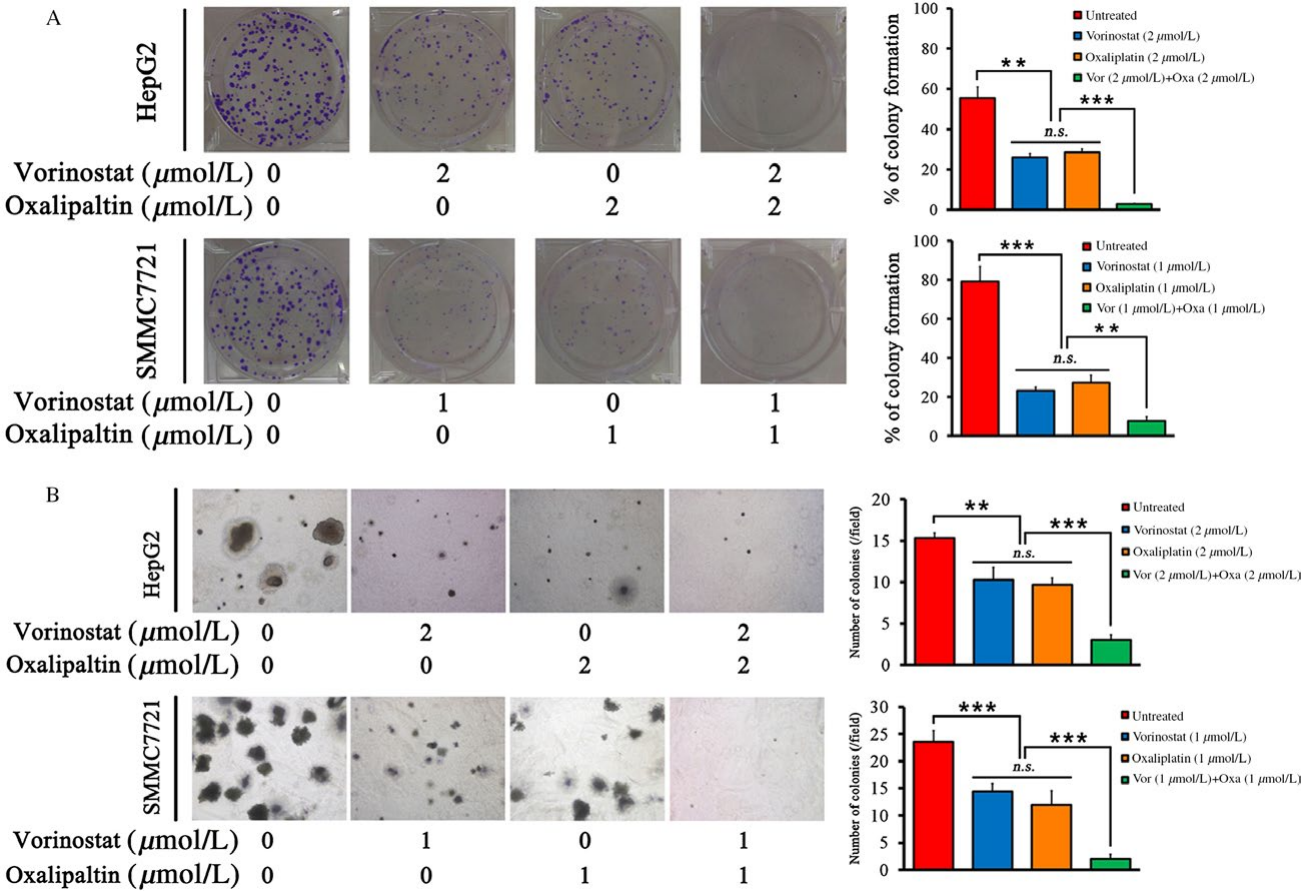
Combination of vorinostat and oxaliplatin inhibited tumorigenicity in vitro. (A) Colony formation assay. HepG2 cells (500 cells/well) were treated with 2 *μ*mol/L vorinostat and/or 2 *μ*mol/L oxaliplatin in six‐well plates for 48 h. SMMC7721 (300 cells/well) were treated with 1 *μ*mol/L vorinostat and/or 1 *μ*mol/L oxaliplatin in six‐well plates for 48 h. The wells were stained and images were acquired at day 14. The percentage of cells that formed colonies was calculated. (B) Soft agar assay. HepG2 cells (5000 cells/well) were treated with 2 *μ*mol/L vorinostat and/or 2 *μ*mol/L oxaliplatin in six‐well plates for 14 days. SMMC7721 (5000 cells/well) were treated with 1 *μ*mol/L and/or 1 *μ*mol/L oxaliplatin in six‐well plates for 14 days. The average number of colonies in a field was calculated. ***P < 0.001, **P < 0.01.

To investigate the effect of vorinostat and oxaliplatin on the tumorigenicity of HCC cells, colony formation assays and soft agar assays were performed in HepG2 and SMMC7721 cell lines. Vorinostat or oxaliplatin reduced both the number and size of colonies. Moreover, combination with the two agents further attenuated the tumorigenicity compared with single‐drug treatment (Fig. 2A and B).

### Vorinostat and oxaliplatin trigger cell cycle arrest and apoptosis in HCC cells

**Figure 3:**
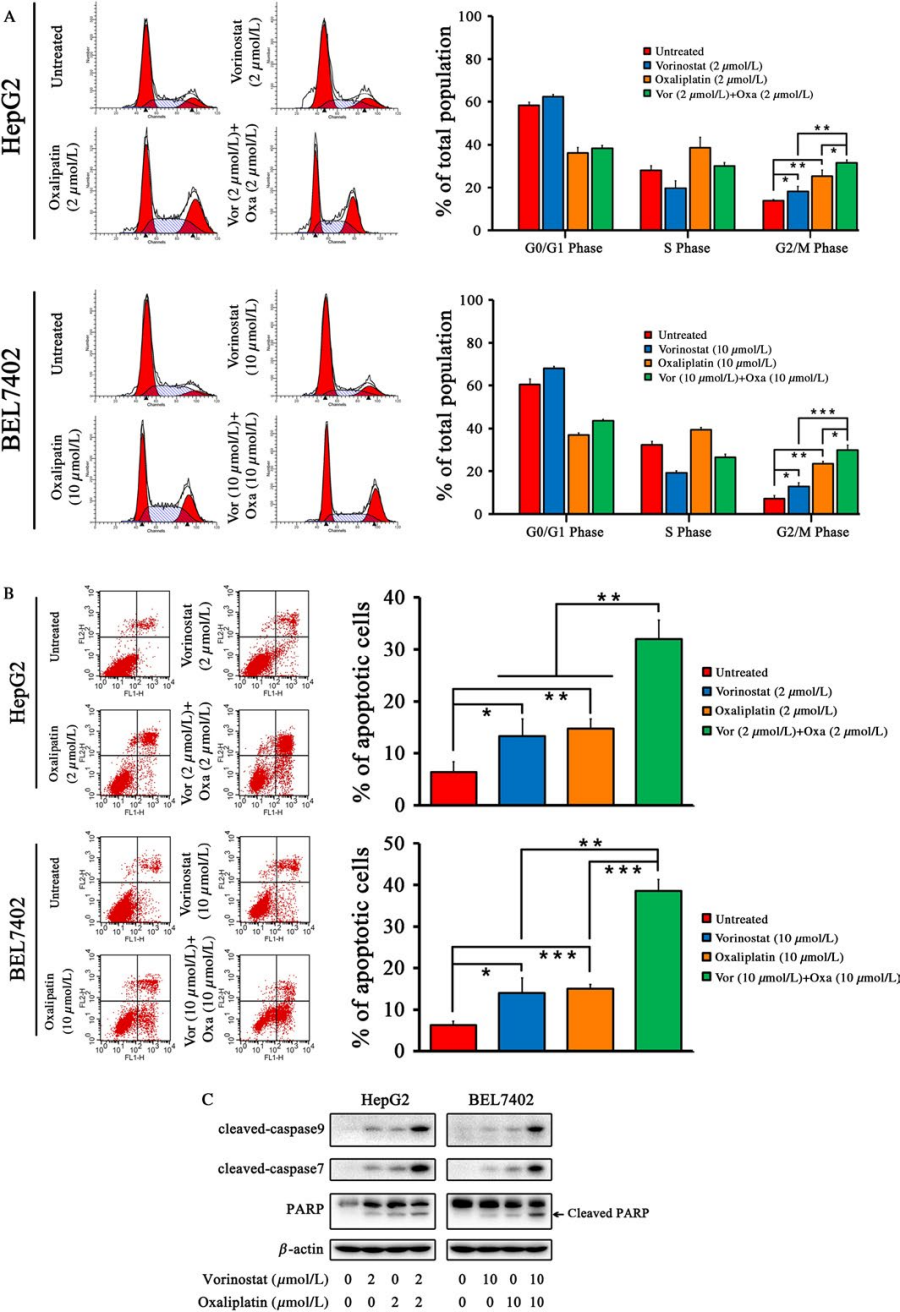
Combination of vorinostat and oxaliplatin induced cell cycle arrest and caspase‐dependent apoptosis in HCC cells. (A) Cell cycle analysis after 48‐h treatment with vorinostat and/or oxaliplatin in HepG2 and BEL7402 cells and the proportion of cells in each cell cycle phase. (B) Apoptosis analysis after 48‐h treatment with vorinostat and/or oxaliplatin and the apoptotic rate of cells in HepG2 and BEL7402 cells. (C) Western blot analysis of the expression of cleaved‐caspase 9, cleaved‐caspase 7, and PARP including cleaved‐PARP after 48‐h drug treatment. HepG2 cells were treated with 2 *μ*mol/L vorinostat and/or 2 *μ*mol/L oxaliplatin and BEL7402 cells were treated with 10 *μ*mol/L vorinostat and/or 10 *μ*mol/L oxaliplatin. ***P < 0.001, **P < 0.01, *P < 0.05.

After vorinostat treatment, the number of cells in G0/G1 and G2/M phases was increased with an accompanying reduction in the number of cells in S phase compared with the control group. After oxaliplatin treatment, the number of cells in S and G2/M phases increased with a concomitant decrease in the numbers of cells in G0/G_1_ phase. Cotreatment with the two agents induced a greater proportion of cells in G2/M phase compared with either drug used in isolation (Fig. 3A). Both vorinostat and oxaliplatin induced apoptosis in HepG2 and BEL7402 cells and coadministration significantly augmented the apoptotic rate compared with either vorinostat or oxaliplatin used alone (Fig. 3B). Western blot analysis showed that the expression of apoptotic proteins including cleaved‐PARP, cleaved‐caspase 7, and cleaved‐caspase 9 was increased after combined treatment with the two agents (Fig. 3C).

### Vorinostat enhances the anticancer effect of oxaliplatin in an animal model

**Figure 4:**
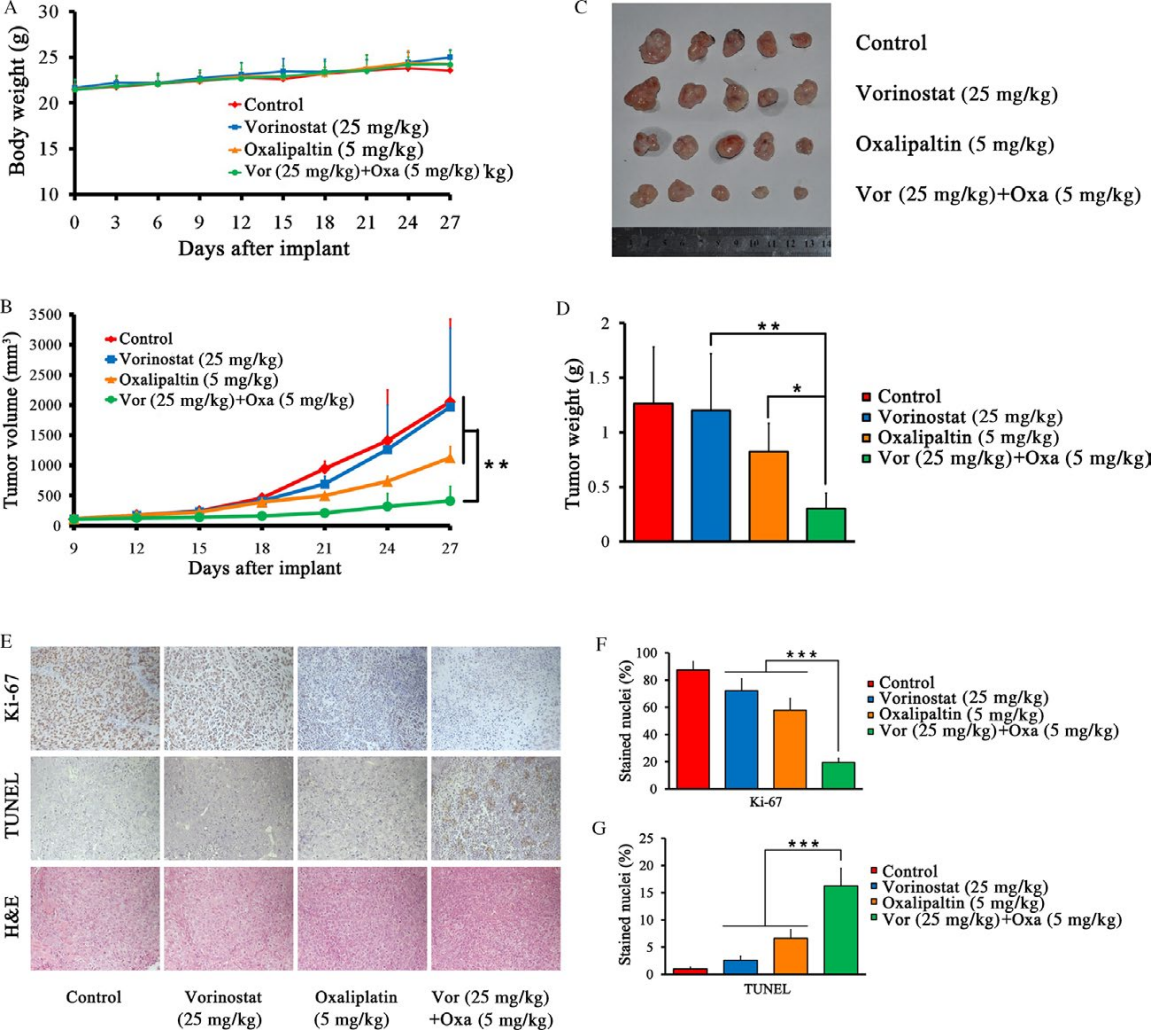
Combination of vorinostat and oxaliplatin inhibited tumorigenicity in animal model. (A) Body weight curve for different groups. (B) Tumor growth curve during the animal model study. At day 9 after injection of cancer cells, drug treatment was initiated and the tumor volume was calculated every third day. Vorinostat (25 mg·kg^−1^) was injected intraperitoneally everyday and oxaliplatin (5 mg·kg^−1^) was injected intraperitoneally twice a week. (C) Photographs of HCC xenografts of all groups on the day of sacrifice. (D) Tumor weights of each group. (E) Representative images of Ki‐67, TUNEL, and hematoxylin and eosin (H&E) staining for growth and apoptosis analysis (200×). (F, G) Proliferation index as determined by the proportion of Ki‐67‐stained nuclei and the apoptotic index as determined by the proportion of TUNEL‐stained nuclei in different groups. ***P < 0.001, **P < 0.01, *P < 0.05.

We next investigated the effect of vorinostat and oxaliplatin on HCC cell growth in vivo. In this study, animal body weight was determined as an indicator of toxicity. The results of body weight curve showed no significant loss in body weight in all experimental groups (Fig. 4A). Drug treatment started when the tumor size grew to approximately 100 mm^3^ at day 9. Mice were sacrificed at day 27. Neither vorinostat (25 mg·kg^−1^ everyday) nor oxaliplatin (5 mg·kg^−1^ twice a week) alone significantly decreased HCC cell proliferation compared with the control group. However, the combination of the two drugs induced a significant decrease in HCC tumor weight and size (Fig. 4B–D). The proportion of Ki‐67‐positive cells, representing the proliferation index, was significantly reduced and the proportion of TUNEL‐positive cells, representing the apoptotic index, was significantly elevated after cotreatment with vorinostat and oxaliplatin (Fig. 4E–G).

### Vorinostat suppresses the expression of BRCA1 in HCC cells

**Figure 5:**
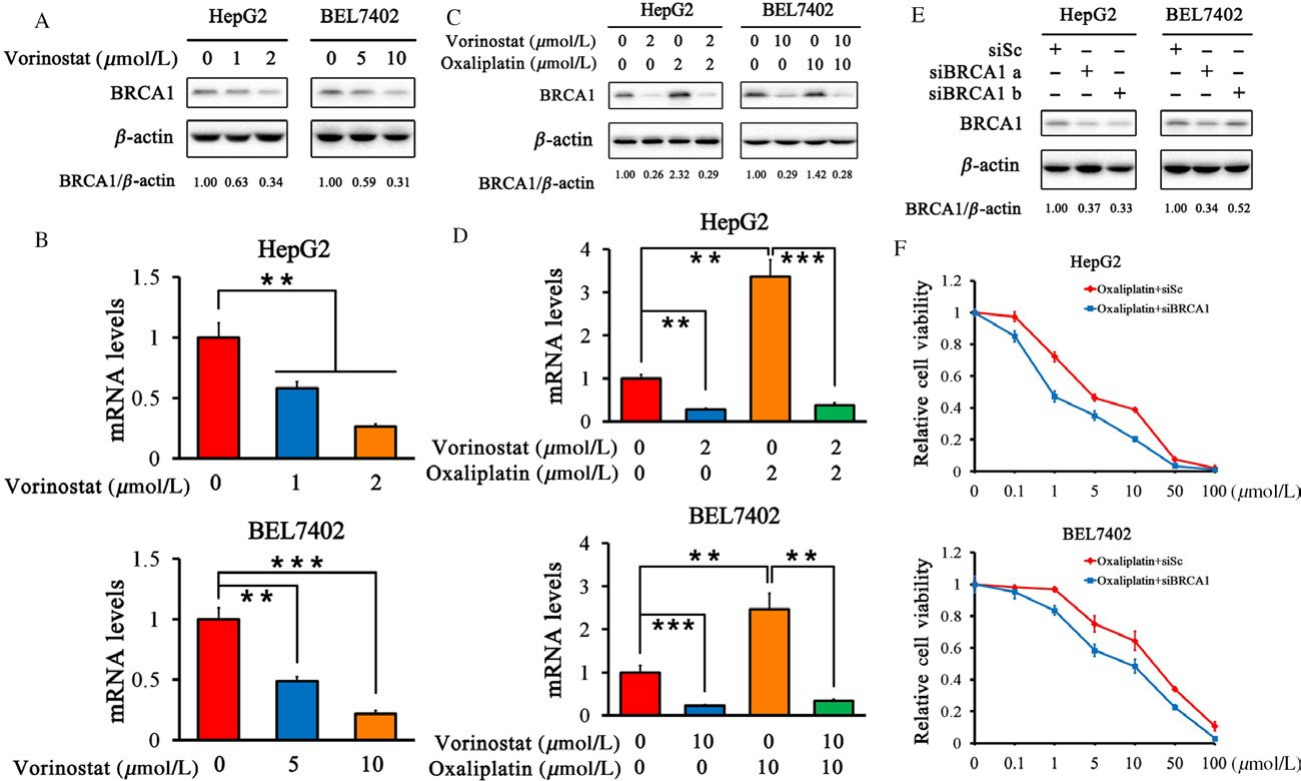
Vorinostat suppressed the expression of BRCA1 induced by oxaliplatin in HCC cells. (A and B) Protein and mRNA expression of BRCA1 by western blotting and qPCR after treatment with vorinostat. HepG2 cells were treated with vorinostat (1 *μ*mol/L and 2 *μ*mol/L). BEL7402 cells were treated with vorinostat (5 *μ*mol/L and 10 *μ*mol/L). (C and D) Protein and mRNA expression of BRCA1 by western blotting and qPCR after treatment with vorinostat and/or oxaliplatin. HepG2 cells were treated with 2 *μ*mol/L vorinostat and/or 2 *μ*mol/L oxaliplatin. BEL7402 cells were treated with 10 *μ*mol/L vorinostat and/or 10 *μ*mol/L oxaliplatin. (E) Inhibition of BRCA1 expression by siRNA was confirmed by western blotting. (F) Relative viability curves of HCC cells after treatment with oxaliplatin alone or with BRCA1 inhibition. Quantification of each band by densitometry was performed and the BRCA1/*β*‐actin ratio was indicated. ***P < 0.001, **P < 0.01.

We examined the effect of 48‐h treatment with vorinostat and oxaliplatin on the expression of BRCA1 in HCC cells in vitro. Western blotting showed that vorinostat downregulated the protein expression of BRCA1 in a dose‐dependent manner (Fig. 5A). The mRNA level of BRCA1 was also decreased by vorinostat using quantitative PCR (qPCR) (Fig. 5B). Oxaliplatin increased the protein and mRNA expression of BRCA1, as determined by western blotting and qPCR (Fig. 5C and D). This effect was partially reversed by the addition of vorinostat (Fig. 5C and D). BRCA1 expression was specifically inhibited by siRNA, as verified by western blotting (Fig. 5E). The relative cell viability of HCC cells was much lower after BRCA1 inhibition and exposure to oxaliplatin than after treatment with oxaliplatin alone (Fig. 5F).

## Discussion

Our study evaluated the combined effect of vorinostat and oxaliplatin on HCC. Both vorinostat and oxaliplatin alone attenuated the proliferation of HepG2, SMMC7721, and BEL7402 cells. When the two agents were combined, the CI values were less than 1 in all three cell lines, indicating a synergistic effect of the two agents, and all DRI values were greater than 1, demonstrating reduced dose and toxicity for the combination. Cotreatment with vorinostat and oxaliplatin induced G2/M phase arrest as shown by flow cytometry. Vorinostat or oxaliplatin used in isolation triggered apoptosis as evidenced by flow cytometry and western blotting and coadministration had a synergistic effect. The combination of vorinostat and oxaliplatin significantly inhibited HCC tumorigenicity in vitro and in vivo.

Sorafenib is a noncurative therapy that improves the survival of patients with advanced HCC 45, 46, 47. However, it is not widely used in Asia because of the vast expense and potentially increased adverse events in the Asian population 48, 49, 50. Therefore it is necessary to identify novel agents for testing in clinical trials.

Histone acetylation is a key factor in the regulation of chromatin accessibility as determined by the nuclear distribution of microinjected fluorescein‐labeled dextrans assessed by measuring the fluorescein‐dextran sizes combined with image correlated spectroscopy 19, 51. After treatment with HDAC inhibitors, acetylation of the core histones is increased, the N‐terminal tails of histones are removed, the interplay between histone and chromatin is loosened, and the DNA configuration becomes open and much more accessible to transcriptional factors and DNA‐targeting drugs 52, 53, 54.

**Figure 6:**
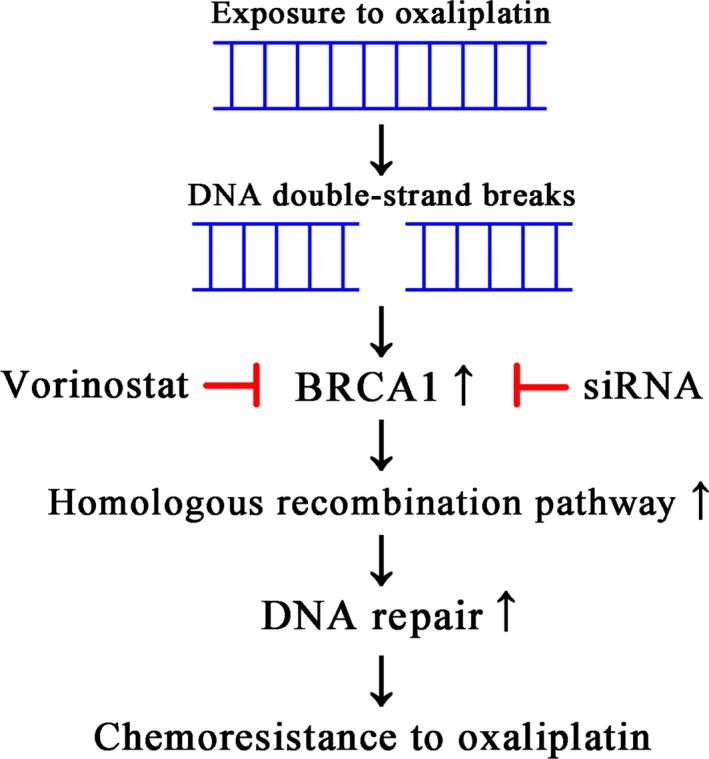
Schematic representation of the mechanism underlying chemoresistance to oxaliplatin.

Chemoresistance to oxaliplatin is a very challenging problem to circumvent. DNA damage repair is the most important mechanism underlying tumor resistance to oxaliplatin and occurs through four basic mechanisms, including DNA double‐strand break (DSB) repair. Homologous recombination (HR) repair is the most important pathway of DSB repair 55, 56 and breast cancer susceptibility gene 1 (BRCA1) is a key member of the HR pathway 57, 58. BRCA1 was identified in 1990 59. It is frequently mutated in ovarian and breast carcinomas and is involved in multiple cellular functions such as DNA repair, transcription, and recombination 60, 61. BRCA1 deficiency is associated with increased sensitivity to oxaliplatin 62. In this study, we found that BRCA1 expression correlated with resistance to oxaliplatin (Fig. 6). The expression of BRCA1 was elevated after oxaliplatin treatment and BRCA1 inhibition increased chemosensitivity to oxaliplatin.

HDAC inhibitors can attenuate DNA damage repair and suppress the expression of BRCA1 in many tumors including cervical, head and neck cancer, prostate, ovarian, and breast cancers 63, 64, 65, 66, 67, 68. In this study, we showed that BRCA1 expression was upregulated by oxaliplatin exposure alone, whereas vorinostat downregulated BRCA1 expression in the presence or absence of oxaliplatin in HCC cells (Fig. 6).

Vorinostat, a broad‐spectrum HDAC inhibitor, can inhibit many histone deacetylases including HDAC1 and HDAC2. HDAC1 and HDAC2 have been proven to cooperate in regulating BRCA1 transcript and protein expression in acute myeloid leukemia (AML) cells 69. It has been reported that BRCA1 associates with some components of the histone deacetylase complex and that the carboxylterminal domain (BRCT) of BRCA1 interacts with the histone deacetylases HDAC1 and HDAC2 61. We presume that vorinostat suppresses HDAC1 and HDAC2, which interact with and inhibit BRCA1. This might be one of the mechanisms underlying the regulation of BRCA1 expression by vorinostat, and requires further research.

Taken together, the combination of vorinostat and oxaliplatin has a synergistic effect in HCC cells. Their coadministration attenuates the growth of cells, induces G2/M phase arrest, causes apoptosis, and decreases tumorigenicity both in vitro and in vivo. Vorinostat enhances the anticancer effect of oxaliplatin on HCC cells by suppressing the expression of BRCA1 that is induced by oxaliplatin. Vorinostat is a potent chemosensitizer of oxaliplatin, which is a DNA‐targeting agent. Cotreatment with vorinostat and oxaliplatin may be a promising novel strategy against advanced HCC.

## Conflict of Interest

All the authors declared no competing interests.
